# The *dnd* RNA Identifies Germ Cell Origin and Migration in Olive Flounder (*Paralichthys olivaceus*)

**DOI:** 10.1155/2015/428591

**Published:** 2015-05-28

**Authors:** Xueying Wang, Qinghua Liu, Yongshuang Xiao, Yang Yang, Yanfeng Wang, Zongcheng Song, Feng You, Hao An, Zhizhong Xiao, Shihong Xu, Daoyuan Ma, Jun Li

**Affiliations:** ^1^Center of Biotechnology R&D, Institute of Oceanology, Chinese Academy of Sciences, Qingdao 266071, China; ^2^University of Chinese Academy of Sciences, Beijing 100049, China; ^3^Weihai Shenghang Aquatic Product Science and Technology Co. Ltd., Weihai 264200, China; ^4^Key Laboratory of Experimental Marine Biology, Institute of Oceanology, Chinese Academy of Sciences, Qingdao 266071, China

## Abstract

The present study obtained a germ cell-specific marker dead end (*dnd*) in olive flounder (*Paralichthys olivaceus*) named* Podnd*. The tissue-specific expressions of* Podnd* transcripts were present in testis and ovary but were not detectable in other somatic tissues detected. SISH showed that* Podnd* expressed only in germ cells at different developmental stages but not in surrounding somatic cells. The expression of* Podnd* during embryonic development at 16 different stages revealed that the relative expression of* Podnd* transcript fluctuated at a high level in the cleavage stages, gradually decreased through subsequent development, and reached the lowest at late gastrula stage till it was nearly undetectable. The* Podnd* transcripts localization and migration were similar to zebrafish. Further research on the specification migration mechanism of PGCs and the role of germ cell during gonadal development in olive flounder would improve our understanding of germline development.

## 1. Introduction

Primordial germ cells (PGCs) are a small population of cells segregated from other cell lineages during embryogenesis. Germline development originates from the formation of PGCs, which migrate from the position where they are specified toward the presumptive genital ridge [1]. Germ cells whose unique role is to transmit genetic information to the next generations can only be detected after gastrula stage by histological methods, until germ cell-specific marker emerged, expressed specifically in PGCs, such as dead end (*dnd*) [2],* vasa* [3], and* nanos* [4]. The PGCs development has been attracting more attention, as the specific markers were applied to track PGCs origination and migration during embryogenesis.

Dead end (*dnd*) is a vertebrate germ cell-specific marker that was first identified as a gene encoding an RNA-binding protein essential for zebrafish PGC migration and survival [2]. Similarly, knockdown of the* dnd* gene in mouse,* Xenopus*, also caused PGCs loss and abnormal migration. The* dnd* mRNA expression showed sex-specific, such as female-specific, expression in* Xenopus* [5] and bisexual expression in chicken [6], medaka [7], and turbot [8–10]. In addition, the development of PGCs and their role in normal gonadal development and differentiation are important in mouse, chicken [6], and fishes [11–13]. The different expression pattern of* dnd* between male and female among organisms may suggest its diverse function during evolution.

It has been reported that germplasm specification from cleavage stage to presumptive genital ridge region is different among teleost fishes. In zebrafish, at cleavage stage, the* vasa*,* dnd* mRNA was localized to the edge of cleavage furrows [14, 15], while, in medaka, the* vasa* mRNA localization function was lost [16]. Furthermore, the migration path of PGCs toward the genital ridge appeared to be varied by the position specified for teleost [17]. In zebrafish [18], PGCs actively migrated by attraction towards an intermediate target and then moved posterior-ward along the border of the trunk mesoderm to settle at the junction of the yolk extension, whereas, in ukigori [19], PGCs migrated in the dorsal direction and from both sides seemed to be mingled on the endoderm and were redistributed to both sides of the gut. Recently, as artificial breeding technology breakthrough, research on germ cell development and differentiation in marine fishes has received increasing research focus.

Olive flounder (*Paralichthys olivaceus*) is an economically important marine flatfish species for aquaculture and females grow faster than males [20]; however, limited information on germ cell origination, migration, and differentiation has been reported. So, germ cell markers are needed to identify and to research on germline development for sex control. The aims of this study were (1) to isolate and identify a germ cell-specific marker* dnd*-like gene (*Podnd*) in olive flounder; (2) to investigate its tissue-specific and embryonic expression levels by real-time quantitative PCR (qRT-PCR); (3) to detect* Podnd* expression pattern in gonadal germ cells for both sexes; and (4) to trace the germ cell lineage origination and migration during embryogenesis by whole mount in situ hybridization (WISH).

## 2. Materials and Methods

### 2.1. Fish and Embryo Collection

Mature olive flounder used in this study was obtained from Oriental Ocean Sci-Tech Co., Ltd. (Shandong Province, China). Tissue samples from ten fish of 42.50 cm–45.23 cm total length and 1.23 kg–1.54 kg body weight (mean ± standard deviation) were rapidly excised after anesthesia with 0.05% solution of ethyl 3-aminobenzoate methanesulfonate (Sigma-Aldrich, Shanghai, China). The tissue samples included heart, brain, liver, gut, stomach, kidney, spleen, muscle, gill, testis, and ovary. Fertilized eggs were obtained by artificial insemination and cultured at 16°C ± 0.5°C in fresh seawater at Shenghang Sci-Tech Co., Ltd. (Weihai, Shandong Province, China).

Tissue and embryos samples were stored in liquid nitrogen for RNA extraction. For WISH, embryos were fixed in 4% paraformaldehyde (Sigma-Aldrich) in PBS overnight and stored at −20°C in PBS with 50% formamide (Sangon, Shanghai, China). For section in situ hybridization (SISH), ovary samples were fixed in 4% paraformaldehyde in PBS overnight and embedded in paraffin after dehydration in a series of ethanol baths and clearance by xylene.

### 2.2. cDNA Cloning of Olive Flounder* dnd* (*Podnd*) Homologue

Total RNA was extracted from the ovary of olive flounder using RNA fast 200 (Fastagen Biotech, Shanghai, China), according to the manufacturer's instructions, and all total RNA was dissolved in 25 *μ*L RNase-free water. The first-strand cDNAs were synthesized using a TransScript one-step gDNA removal and cDNA synthesis SuperMix (TransGen Biotech, Beijing, China) according to the manufacturer's instructions. The cDNA fragment of the* Podnd* gene was amplified by RT-PCR with primers (*Podnd* F1 and* Podnd* R1) designed according to the highly conserved regions of* dnd* homologues from various fish species (Table 1). The PCR was performed in 20 *μ*L reaction volume with primers* Podnd* F1 and* Podnd* R1 containing 0.2 *μ*L TransTaq HiFi DNA polymerase, 1.6 *μ*L 5 mM dNTPs, 0.2 *μ*L (10 *μ*M) forward primer and reverse primer, 0.5 *μ*L cDNA, 2 *μ*L 10× TransTaq HiFi buffer, and 15.3 *μ*L ddH_2_O. Amplification was carried out using a XP-D thermal cycle (Bioer, Beijing, China) and the reaction proceeded as follows: denaturation at 94°C for 5 minutes; 35 cycles of amplification at 94°C for 30 seconds, 52°C for 30 seconds, 72°C for 1 minute, and finally an additional elongation at 72°C for 5 minutes. The resultant cDNA fragments were cloned in a plasmid vector pMD^TM^18-T (Takara, Dalian, China) and sequenced.

Subsequently, 5′ and 3′ rapid amplification of cDNA ends (RACE) were performed to clone the full-length coding sequence with a SMARTer RACE cDNA Amplification Kit (Clontech, Mountain View, CA, USA) according to the manual. The gene-specific primers for 5′-RACE (*Podnd*-5RACE-1 and* Podnd*-5RACE-2) and 3′-RACE (*Podnd*-3RACE-1 and* Podnd*-3RACE-2) were also showed in Table 1. PCR was performed using denaturation at 94°C for 5 minutes; 35 cycles of amplification at 94°C for 30 seconds, 62°C for 45 seconds, 72°C for 2 minutes; and an additional elongation at 72°C for 5 minutes. Each PCR product was separated by electrophoresis on 1% agarose gel. The fragments were purified and subcloned into pMD^TM^18-T Vector (TaKaRa, Dalian, China) for sequencing. The positive clones were obtained by antibiotic selection and the inserts were sequenced at Sangon (Shanghai, China). Sequences were analysed by the Lasergene software package (DNAStar, Madison, USA) and examined against the GenBank database.

### 2.3. Phylogenetic Analysis

A homology search of the nucleotide and deduced amino acid sequences of* Podnd* were confirmed using the National Center for Biotechnology Information Website (http://www.ncbi.nlm.nih.gov/). The deduced amino acid sequences were aligned using the AlignX program in Vector NTI suite 11.5 software package. The phylogenetic tree was constructed by Mega 6 software with neighbor-joining method. The branching reliability was tested via bootstrap analysis of 1000 replicates.

### 2.4. In Situ Hybridization and Histology

Localization of* Podnd* mRNA was analyzed by WISH and paraffin sections of ovary, as previously described by Lin et al. [21]. Sense and antisense* Podnd* probes were individually synthesized using the DIG RNA Labeling Kit (SP6/T7) (Roche, Mannheim, Germany) following the manufacture's instruction. The 724 bp* dnd* PCR product (529–1253) was subcloned into pGEM-T Easy Vector (Promega) and transcribed in vitro to obtain the probes. Paraffin sections of 1-day larva hybridized with* Podnd* antisense probe were applied to identify the precise location of PGCs. Meanwhile, for SISH, paraffin sections (7 *μ*m) were cut, dewaxed by xylene, and rehydrated through an ethanol series. Before hybridization, sections were washed with PBST, refixed with 4% paraformaldehyde, and permeabilized with 0.2 M HCl, 10 *μ*g/mL proteinase K (Sangon). The other procedures for washes and detection of DIG were similar to the WISH.

For histology, the ovaries were fixed in Bouin's fixative for 24 h, dehydrated in an ascending ethanol series, cleared with xylene, and embedded in paraffin, according to standard paraffin-embedding methods. Tissues were cut into 7 *μ*m thick sections and stained with hematoxylin and eosin (H&E). For WISH, SISH, and histology, the photographs were taken using a Nikon E50i microscope with Nikon DS-Fi imaging system.

### 2.5. Real-Time PCR

Total RNA was extracted from embryos and adult tissues including heart, brain, liver, gut, stomach, kidney, spleen, muscle, gill, testis, and ovary. All assays were carried out in triplicate for each stage of embryo independently. The number of embryos in each triplicate was about 50. The biological replicates and technical replicates for each tissue were three independently. The expressions of* Podnd* in different tissues and embryonic samples were analyzed by qRT-PCR using SYBR Green detection method in an ABI 7300 real-time PCR instrument (Applied Biosystems, Foster City, CA, USA).

The results were analyzed by comparative Ct method.* Podnd* expression was normalized against *β*-actin, generating a ΔCt (Ct(*Podnd*) − Ct(*β-actin*)). According to the equation, 2^−ΔΔCt^, relative expression was calculated. During tissue-specific expression, gut was taken as a calibrator and during embryogenesis 16-cell stage was taken as a calibrator.

### 2.6. Statistical Analysis

Statistical analysis was carried out using the software package SPSS 19.0 for Windows (SPSS Inc., Chicago, IL, USA), and data were expressed as mean ± SD (*P* < 0.05). qRT–PCR data was statistically analyzed by one-way ANOVA followed by a Tukey test, respectively. All assays were carried out in triplicate for each stage independently and *P* < 0.05 denoted statistically significant difference.

## 3. Results

### 3.1. Molecular Characterization of* Podnd*

**Figure 1:**
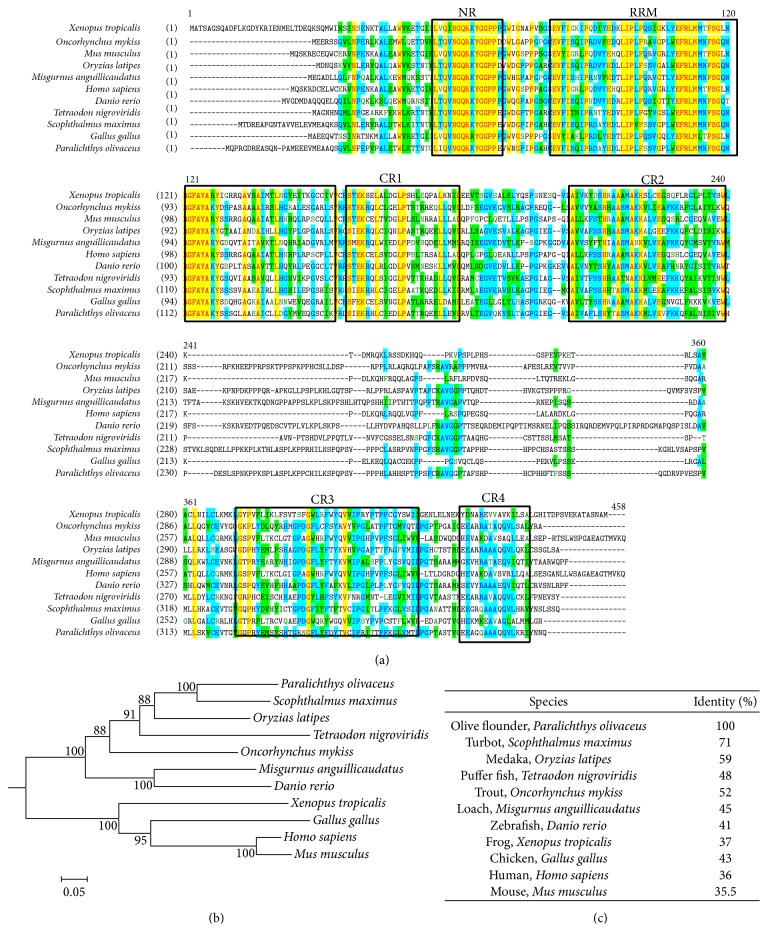
Comparison of DND proteins. (a) Multiple alignment was carried out using AlignX program. The conserved regions are indicated in frame. NR: N-terminal region; RRM: RNA recognition motif; CR1–4: C-terminal regions. (b) Phylogenetic tree of DND proteins. It was established by MEGA6 with neighbor-joining method. The numbers under the node indicate the bootstrap value as percentages obtained for 1000 replicates. (c) Comparison of the identities of DND homologues between olive flounder and other organisms.

A partial cDNA fragment (176 bp) of* dnd* was isolated from* Paralichthys olivaceus* ovary. The full sequence of* Podnd* was obtained successfully by using 3′- and 5′-RACE. The full length of* Podnd* cDNA is 1446 bp, containing a 1178 bp open reading frame. It encodes a putative protein of 392 amino acids, an 18 bp 5′-untranslated region (UTR), and a 249 bp 3′-UTR. The nucleotide sequence of* Podnd* had been submitted to GenBank (accession number: KP224455). The deduced amino acid sequences were aligned using the AlignX program in Vector NTI suite 11.5 software package (Figure 1(a)).

### 3.2. Phylogenetic Analysis

A phylogenetic tree was constructed with full-length DND sequences by Mega 6 software, using neighbor-joining method with a bootstrap analysis of 1000 replicates (Figure 1(b)). The deduced DND protein sequence was aligned with related protein sequence of other typical vertebrates (Figure 1(c)). The identity of DND to that of other species is 71, 59, 52, 45, 43, 41, 37, 36, and 35.5% identical to the* Scophthalmus maximus* (AGI78910),* Oryzias latipes* (GQ184560),* Oncorhynchus mykiss* (NP_001118133.1),* Tetraodon nigroviridi*s (CAF92275.1),* Misgurnus anguillicaudatus* (BAJ19134.1),* Gallus gallus* (XP_423051.4),* Danio rerio* (NP_997960),* Xenopus tropicalis* (NP_001037899.1),* Homo sapiens* (NP_919225.1), and* Mus musculus* (NP_775559.2).

### 3.3. *Podnd* RNA Expression in Different Tissues and during Embryogenesis by qRT-PCR

**Figure 2:**
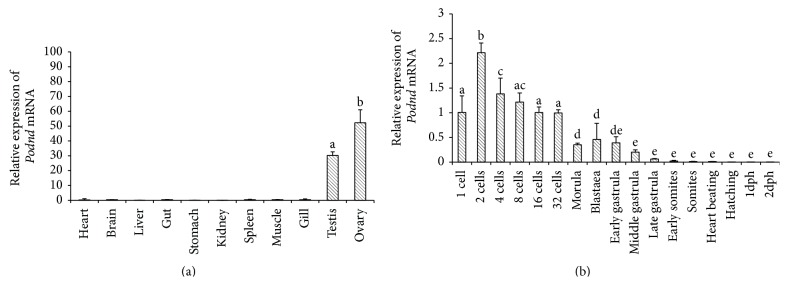
Expression of* Podnd* mRNA determined by qRT-PCR. (a) Tissue-specific expression of* Podnd* mRNA determined by qRT-PCR. (b) Embryonic expression of* Podnd* determined by qRT-PCR.

*Podnd* mRNA expression levels in different tissues including heart, brain, liver, intestine, kidney, spleen, muscle, gill, ovary, and testis of mature olive flounder by qRT-PCR were performed. Total RNA was extracted from tissues of healthy adults, each sample of different developmental stages during embryogenesis, and reversely transcribed. The results showed that tissue-specific expressions of* Podnd* transcripts were present in testis and ovary but were not detectable in other somatic tissues detected (Figure 2(a)). In addition, the expression of* Podnd* transcripts was much higher in the ovary compared with that in the testis.

The expression of* Podnd* during embryonic development at 16 different stages revealed that the relative expression of* Podnd* transcript fluctuated at a high level in the cleavage stages (Figure 2(b)). The highest level presented at the 2-cell stage, gradually decreased through subsequent development, and reached the lowest at late gastrula stage till it was nearly undetectable.

### 3.4. Localization of* Podnd* RNA in Gonad by Tissue Section In Situ Hybridization

**Figure 3:**
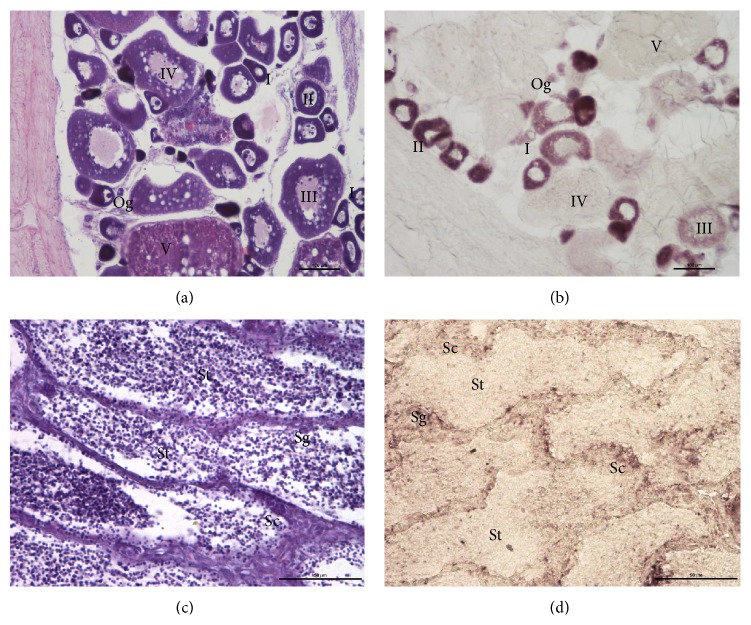
Distribution of* Podnd* transcripts in gonad germ cells by SISH. (a) Paraffin sections of ovary were stained by HE; (b) paraffin sections of ovary were hybridized with* Podnd* antisense probe. On ovarian sections, the strongest signal was present in certain oogonia and oocytes at stage II (diffused in the ooplasm), whereas, difficult to discern in oocytes at stage V,* Podnd* transcripts were diffused throughout the ooplasm. (c) Paraffin sections of testis were stained by HE; (d) paraffin sections of testis were hybridized with* Podnd* antisense probe.* Podnd* signal was strong in spermatogonia, weak in spermatocytes, and absent in spermatids. Og: oogonia; I-II: previtellogenic oocytes; III: early vitellogenic oocytes; IV: midvitellogenic oocytes; V: late vitellogenic oocytes; Sg: spermatogonia; Sc: spermatocytes; St: spermatids. Scale bar: 100 *µ*m ((a)-(b)), 50 *μ*m ((c)-(d)).

Tissue section in situ hybridization was carried out to investigate the* Podnd* expression pattern of adult gonad. SISH showed that* Podnd* expressed only in germ cells at different developmental stages but not in surrounding somatic cells. In ovarian sections, the strongest signal was present in certain oogonia and oocytes at stage II (diffused in the ooplasm) whereas it was difficult to discern in oocytes at stage V (Figure 3(b)). In testis, obvious signal was present in spermatogonium, weaker in spermatocyte, and undetectable in spermatid and spermatozoon (Figure 3(d)).

### 3.5. Spatial Localization of* Podnd* RNA during Embryogenesis

**Figure 4:**
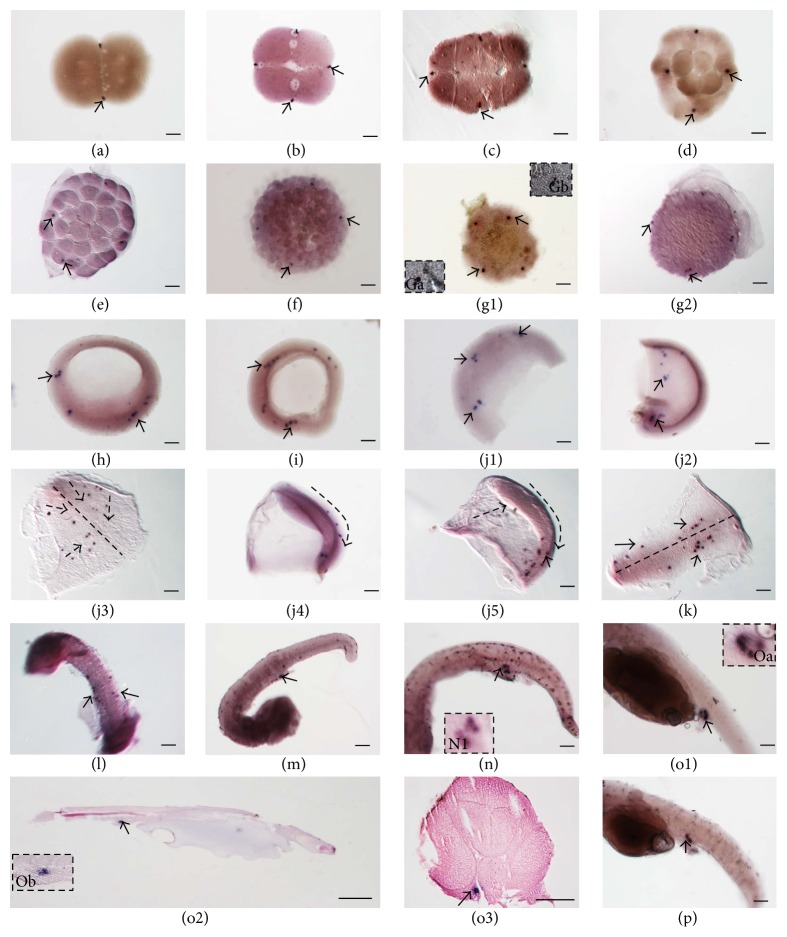
Distribution of* Podnd* transcripts from 2-cell stage to 2 days after hatching. Embryos were hybridized with* Podnd* antisense probe. Black arrows indicate* Podnd* transcript aggregated regions (dark purple). Dotted straight line indicates embryonic body axis. Dotted arrows indicate PGC migration direction. (a) 2-cell stage:* Podnd* transcripts are concentrated at the distal edge of the 1st cleavage furrow; (b) 4-cell stage:* Podnd* signals are located as four spots at the distal edge of the 1st and 2nd cleavage furrows; (c) 8-cell stage:* Podnd* transcripts are still at the distal edge of the first two cleavage furrows; (d) 16-cell stage:* Podnd* transcripts are migrating from cleavage furrows to the edge of cells, respectively; (e) 32-cell stage:* Podnd*-positive spots are distributed into four cells, respectively; (f) morula stage:* Podnd* transcripts are still in four cells; ((g1) and (g2)) blastula stage:* Podnd*-positive cells increase and are located adjacent to each other; ((Ga) and (Gb)) magnification of the boxed region in (g1), respectively; (h) top view of early gastrula stage:* Podnd*-positive cells are located in the marginal region of blastoderm. Black arrowheads: (i) top view of mid gastrula stage:* Podnd*-positive cells are located at germ ring; ((j1) and (j2)) dorsal and lateral view of mid and late gastrula stage, respectively:* Podnd*-positive cells move towards the dorsal side, and some cells still lie in the germ ring region; ((j3)–(j5)) lateral view of late gastrula stage, respectively. (j3)* Podnd*-positive cells scattered in anterior of the trunk mesoderm appeared to align along anterior of the body axes from being randomly oriented, (j4)* Podnd*-positive cells moved posterior-ward, and (j5)* Podnd*-positive cells aligned along both sides of the body axes in posterior of the trunk region. (k) Early somite stage:* Podnd*-positive cells aligned along both sides of the body axes. (l) Somite stage:* Podnd*-positive cells moved outward and aligned as two lines along both sides of the embryonic body in the trunk region; (m) heart beating stage: the* Podnd*-positive cells formed clusters above the hind gut in the presumptive genital ridge; (n) hatching stage:* Podnd*-positive cells cluster under the genital ridge, which are above the posterior end of the hind gut and below the somites on either side of the embryonic body. ((o1) and (p)) 1-2 d after hatching:* Podnd*-positive cells cluster in the presumptive genital ridge region at the posterior dorsal sides of gut; (o2) longitudinal section of 1 d larva hybridized with* Podnd* antisense probe:* Podnd*-positive cells cluster in the presumptive genital ridge region at the posterior dorsal sides of gut. (o3) Cross section of 1 d larva hybridized with* Podnd* antisense probe: the* Podnd*-positive cells still aligned at the genital ridge which was at the dorsal sides of the hind gut, ventral side of notochord. Scale bar: 100 *µ*m.

**Figure 5:**
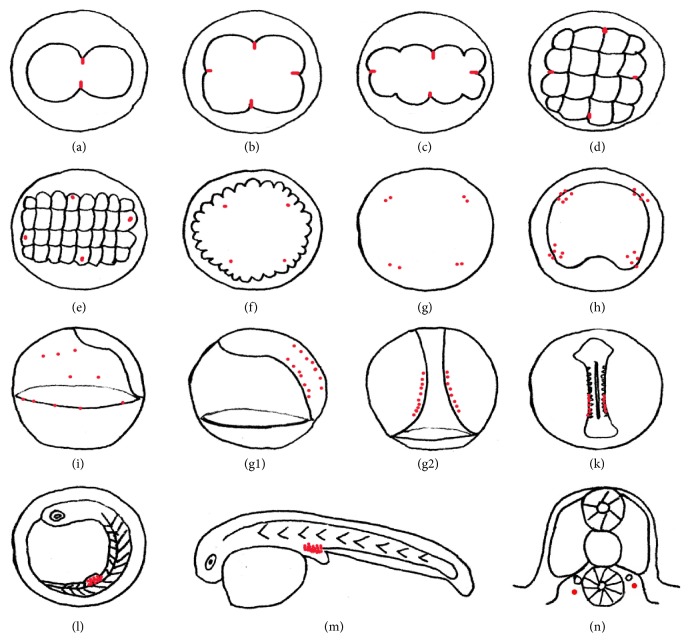
Schematic summary of* Podnd* transcripts distribution during embryogenesis in olive flounder. Red spots indicate* Podnd* signals. (a) 2-cell, (b) 4-cell, (c) 8-cell, and (d) 16-cell stages. ((a)–(d))* Podnd* are located at the end of the first, second, and third cleavage furrows, (e) 32-cell stage: the signals aggregated into four single blastomeres, (f) morula stage, (g) blastula stage: the* Podnd*-positive cells increased from one to two and the sister cells are located adjacent to each other at every positive area, (h) early gastrula:* Podnd* mRNA mainly localized at the marginal region of the blastoderm, (i) mid gastrula stage: some* Podnd*-positive cells are located at the germ ring and the others moved towards to the dorsal side, ((g1) and (g2)) late gastrula stage, (g1) side view and (g2) top view:* Podnd*-positive cells aligned along both sides of the body axes in the posterior of the trunk region, (k) somite stage:* Podnd*-positive cells aligned as two lines along both sides of the embryonic body in the trunk region, (l) the* Podnd*-positive cells formed clusters above the hind gut in the presumptive genital ridge, (m) 1-2 d after hatching:* Podnd*-positive cells cluster in the presumptive genital ridge region at the posterior dorsal sides of gut, and (n) cross section:* dnd*-positive cells aligned at the genital ridge which was at the dorsal sides of the hind gut and ventral side of notochord.

The localization of* Podnd* transcripts was investigated by WISH during embryogenesis from 2-cell stage to 2 days after hatching to reveal the origin and migration of PGCs. At the 2-cell stage (Figures 4(a) and 5(a)),* Podnd* mRNA was detected at either end of the first cleavage furrow. At the 4-cell (Figures 4(b) and 5(b)) and 8-cell (Figures 4(c) and 5(c)) stages, mRNA aggregations had split into four clusters which remained located at the end of the first and second cleavage furrows. As the embryo developed to 32-cell stage (Figures 4(e) and 5(e)), the signals aggregated into four single blastomeres and formed presumptive PGCs. At morula stage (Figures 4(f) and 5(f)), the four* Podnd*-positive cells were still located at the margin of blastoderm. During the blastula stage (Figures 4(g1), 4(g2), and 5(g)), the* Podnd*-positive cells increased from one to two and the sister cells were located adjacent to each other at every positive area and then formed PGCs.

As the embryo developed to the early gastrula,* Podnd* mRNA mainly localized at the marginal region of the blastoderm (Figures 4(h) and 5(h)). At the mid gastrula stage, some* Podnd*-positive cells moved towards to the dorsal side (Figures 4(i) and 5(i)). By late gastrula stage (Figures 4(j1)–4(j5) and 5(g1) and 5(g2)),* Podnd*-positive cells scattered in anterior of the trunk mesoderm (Figure 4(j3)) and appeared to align along anterior of the body axes from being randomly oriented. Next, they moved posterior-ward (Figures 4(j4) and 4(j5)). And they moved outward and aligned as two lines to the somite stage (Figures 4(k), 4(l), and 5(k)).

At the heart beating stage (Figures 4(m) and 5(l)), the* Podnd*-positive cells formed clusters above the hind gut in the presumptive genital ridge. Subsequently, at the hatching period (Figures 4(n), 4(c), and 5(m)), the* dnd*-positive cells formed two relatively compact clusters under the genital ridge, which were above the posterior end of the hind gut and below the somites on either side of the embryonic body. Then 1 day after hatching (Figure 4(o1)) and 2 days after hatching (Figure 4(p)), the positive cells still aligned at the genital ridge (Figures 4(o2), 4(o4), and 5(n)), at the dorsal sides of the hind gut, ventral side of notochord.

## 4. Discussion

In the present study, we isolated and characterized a* dnd* homolog gene* Podnd*. The* Podnd* contains RNA recognition motif (RRM) and five conserved regions (NR, CR1–4), with high identity to other DND proteins. N-terminal RNA recognition motive (RRM) and C-terminal ATPase activity within DND mRNA were found very important in PGC development and survival [22, 23]. Phylogenetic trees based on DND amino acid sequences showed that all fish species formed a distinct group separated from those of other vertebrates, and a significant homology was obtained with another flatfish species turbot.

The tissue specificity and SISH revealed that expression of* Podnd* transcripts was exclusively restricted in oogenetic and spermatogenetic but not in somatic cells. The expression level of ovary was much higher than that of testis. The difference in expression between female and male suggested that* Podnd* may play different role in oogenesis and spermatogenesis. In the ovary,* Podnd* expression showed dynamic localization pattern. The signal diffused evenly in the ooplasm in oogonia, stage I and III oocytes, with the strongest signal in stage II, and can not be detected in stages IV and V. The signal should be still there in stage IV and V oocytes, since it is present at the 2-cell stage unless the zygotic genome is already activated at the 2-cell stage. Quantitative RT-PCR of sorted oocytes will allow the transcript abundance to be determined whether the oocytes are too opaque/yolky or the signal is too “diluted” in the large oocyte volume to be visualized in situ. These analyses suggest that the olive flounder, like zebrafish, may use a maternal mode of germ cell specification.

*Podnd* RNA expression by qRT-PCR during embryogenesis revealed that the strongest expression of* Podnd* occurred at two-cell stage and then gradually decreased to the lowest at late gastrula stage till it was nearly undetectable. However, the number of PGCs signals increased with the embryo development. Similar expression pattern was also revealed in zebrafish [2] and medaka [7] by RT-PCR. These phenomena may be due to the somatic cell proliferation speed which is quicker than that of PGCs.

In this study, as revealed by WISH during cleavage stages,* Podnd* mRNA already presented at the two-cell stage and aggregated at both edges of the first, second, and third cleavage furrows at two-eight cell cleavage stages. It was maternal so it was transcribed in oocytes, maternally inherited, and then localized to the furrows. At blastula stage, the number of* Podnd*-positive cells increased from 4 to 8 and the sister cells were located adjacent to each other. Similar results were reported in zebrafish [2] and turbot [8] marked with* dnd*. Meanwhile, the* Podnd* mRNA location pattern also verified its maternal origination.

In olive flounder, the migration pattern of PGCs detected by WISH with* Podnd* mRNA was summarized in Figure 5. Generally, the PGCs movement was similar to that reported in zebrafish but was not identical. PGCs moved dorsally at early gastrula stage, then scattered in anterior of the trunk mesoderm, and appeared to align along anterior of the body axes from being randomly oriented. It seems that there are intermediate targets within the anterior of trunk mesoderm as reported in zebrafish [24]. Detailed information is required by visualizing the actual movement of PGCs around the anterior of trunk during gastrulation. In the final step, PGCs migrated posterior-ward accompanied with outward along body axes, then aggregated together, respectively, and finally settled at the genital ridge above the hind gut, which was along the inner layer of the lateral plate mesoderm. It appeared slightly in more posterior position than that in the zebrafish [18]. The majority of studies about germ cell marker localization during gametogenesis compared with embryogenesis are limited. Thus, comprehensive comparative studies between species are necessary in the future for better understanding of the properties of PGCs.

## 5. Conclusion

The present study identified* dnd* as a germ cell-specific marker and stage-specific expression pattern was observed during gametogenesis in olive flounder. It seems that the origin and migration of PGCs during embryonic development are mainly conserved among teleosts. Further research on the specification migration mechanism of PGCs and the role of germ cell during gonadal development in olive flounder would improve our understanding of germline development.

## Figures and Tables

**Table 1:** Primers used in the study.

Purpose	Primer name	Sequence
Mid	*dnd*F	AATGGCCAGAGGAAGTATGG
	*dnd*R	CCGCTGAAGTTCATCATGAG

RACE	5*dnd*1	ACCTCGCAGTGGGCTCCTGGGATGG
	5*dnd*2	TGATGAAGACCTCGCAGTGGGCTCC
	3*dnd*1	CCTGTTCAGCTCTGTGGGGCCTCTCT
	3*dnd*2	TCTGTGGGGCCTCTCTGGGAGTTTAG

Probe	T*dnd*F	GTTCTGCGTGTGCTGACT
	T*dnd*R	TTGTCTTGGAACAGGGCT

qRT-PCR	*actin*-F	CCTTCACCACCACAGCCGAGAG
	*actin*-R	ATTCCACAGGACTCCATACCGA
	F-*dnd*-F	CCACATCTCGCCCATCATTC
	F-*dnd*-R	GGATTCAACAGGCACTCGGT
